# Diesel Exhaust Particle (DEP)-induced glucose intolerance is driven by an intestinal innate immune response and NLRP3 activation in mice

**DOI:** 10.1186/s12989-023-00536-8

**Published:** 2023-07-03

**Authors:** Angela J. T. Bosch, Theresa V. Rohm, Shefaa AlAsfoor, Andy J. Y. Low, Zora Baumann, Neena Parayil, Faiza Noreen, Julien Roux, Daniel T. Meier, Claudia Cavelti-Weder

**Affiliations:** 1 Department of Biomedicine, University of Basel, Basel, 4031 Switzerland; 2 Swiss Institute of Bioinformatics, Basel, 4031 Switzerland; 3 Clinic of Endocrinology, Diabetes and Metabolism, University Hospital Basel, Basel, 4031 Switzerland; 4 Department of Endocrinology, Diabetology and Clinical Nutrition, University Hospital Zurich (USZ) and University of Zurich (UZH), Zurich, Switzerland; 5 University Hospital Zurich, Rämistrasse 100, Zürich, 8009 Switzerland

**Keywords:** Air pollution, Diesel exhaust particles, Gut exposure, Glucose metabolism, Metabolic disease, Gastrointestinal tract, Innate immune response, Macrophages, NLRP3, IL-1β

## Abstract

**Background:**

We previously found that air pollution particles reaching the gastrointestinal tract elicit gut inflammation as shown by up-regulated gene expression of pro-inflammatory cytokines and monocyte/macrophage markers. This inflammatory response was associated with beta-cell dysfunction and glucose intolerance. So far, it remains unclear whether gut inflammatory changes upon oral air pollution exposure are causally linked to the development of diabetes. Hence, our aim was to assess the role of immune cells in mediating glucose intolerance instigated by orally administered air pollutants.

**Methods:**

To assess immune-mediated mechanisms underlying air pollution-induced glucose intolerance, we administered diesel exhaust particles (DEP; NIST 1650b, 12 µg five days/week) or phosphate-buffered saline (PBS) via gavage for up to 10 months to wild-type mice and mice with genetic or pharmacological depletion of innate or adaptive immune cells. We performed unbiased RNA-sequencing of intestinal macrophages to elucidate signaling pathways that could be pharmacologically targeted and applied an in vitro approach to confirm these pathways.

**Results:**

Oral exposure to air pollution particles induced an interferon and inflammatory signature in colon macrophages together with a decrease of CCR2^−^ anti-inflammatory/resident macrophages. Depletion of macrophages, NLRP3 or IL-1β protected mice from air pollution-induced glucose intolerance. On the contrary, Rag2-/- mice lacking adaptive immune cells developed pronounced gut inflammation and glucose intolerance upon oral DEP exposure.

**Conclusion:**

In mice, oral exposure to air pollution particles triggers an immune-mediated response in intestinal macrophages that contributes to the development of a diabetes-like phenotype. These findings point towards new pharmacologic targets in diabetes instigated by air pollution particles.

**Supplementary Information:**

The online version contains supplementary material available at 10.1186/s12989-023-00536-8.

## Background

Air pollution has emerged as an unexpected risk factor for diabetes in many epidemiological [1–6] and rodent studies [7, 8]. Previously, we found that gut and lung exposures have distinct metabolic outcomes [9]. Lung exposure to air pollutants leads to lung inflammation, hypercholesterinemia and increased liver lipids. Besides also increasing liver lipids, gut exposure to air pollutants specifically impairs beta-cell secretory capacity, potentially instigated by an inflammatory milieu in the gut [9]. Exposure of the gut to air pollutants can occur through mucociliary clearance from the upper airways and contamination of food and water with pollutants [10, 11]. The observed higher incidence of gastrointestinal tract diseases further highlights the clinical significance of air pollutants impacting the gastrointestinal tract [12–14].

The gut constitutes the largest reservoir of immune cells in the body with intestinal macrophages being the most abundant leukocytes in the healthy gut [15]. Intestinal macrophages are not a homogenous cell population, but consist of distinct subpopulations that follow a specific differentiation trajectory [16, 17]. They originate from monocytes that enter the mucosa in a CCR2-dependent manner and differentiate through a series of intermediaries to give rise to mature macrophages. First, they adopt a CCR2^+^ pro-inflammatory phenotype and then gradually lose their inflammatory phenotype to become CCR2^−^ anti-inflammatory/resident macrophages [16, 17]. Intestinal macrophages have been shown to reach into the gut lumen to sample luminal contents [18]. Their physiological role is to shape host-microbiota symbiosis, manage gut inflammation [19–21], cross talk with T cells [22, 23], and facilitate wound repair [24].

Besides the healthy steady state, intestinal macrophage subpopulations have been studied in different disease states. For example, in inflammatory bowel disease (IBD) such as Crohn’s disease and ulcerative colitis, the normal differentiation trajectory is disrupted as intestinal macrophages retain their immature CCR2^+^ pro-inflammatory phenotype instead of differentiating into CCR2^−^ anti-inflammatory/resident macrophages [17, 19, 25]. It has been suggested that the resultant inflammatory shift is due to enhanced monocyte recruitment [26] and/or abnormal macrophage differentiation [19, 27] and potentially mediated by genetic variants [28]. Interestingly, patients with IBD have a 50% higher risk of type 2 diabetes compared to the general population, which cannot be explained by detection bias or corticosteroid exposure [29]. Besides IBD, CCR2^+^ pro-inflammatory intestinal macrophages are also increased in infectious disease mouse models, where they have been linked to beneficial responses in host defense [30, 31]. Additionally, an intestinal immune cell-microbiota crosstalk has been proposed to affect outcomes associated with metabolic syndrome [32]. We recently found a shift towards more CCR2^+^ pro-inflammatory intestinal macrophages in mice fed a high-fat diet (HFD) [33]. Colon-specific macrophage depletion by intrarectal clodronate liposomes improved glucose tolerance, hence demonstrating a link between colon macrophages and glucose metabolism [33]. Likewise, genetic depletion of intestinal macrophages by an epithelial specific Ccl2-/- mouse model was also associated with improved glucose tolerance and insulin sensitivity [34]. These findings of an obesity-related innate immune response in the mouse gut are of high clinical relevance as pro-inflammatory intestinal macrophages were also increased in obese individuals compared to non-obese controls [35]. Thus, air pollution particles reaching the gut could potentially mount a specific immunological response in the gut and thereby mediate a diabetic phenotype.

To our knowledge, the role of intestinal immune cells has not been studied in the context of air pollution-induced diabetes. To study whether air pollution particles exert their metabolic outcomes via immune-mediated pathways, knockout and pharmacological models that target either innate or adaptive immunity can be used. For example, CCR2-/- mice have a defective C-C chemokine receptor 2, which is a cognate MCP-1 receptor and regulates CCR2-dependent monocyte recruitment [36]. An alternative approach to deplete tissue macrophages pharmacologically is by manipulating the colony stimulating factor 1 (CSF1) or its receptor CSF1R, which promote proliferation, differentiation and survival of macrophages [37]. On the other hand, Rag-/- mice can be used to examine the role of adaptive immune cells as these mice lack mature lymphocytes [38]. The aim of our study was to study the immunological changes in the gut upon gavage with diesel exhaust particles (DEP) and – by using the above-mentioned mouse models – to elucidate a potential link between an immune-mediated response and the development of diabetes. A better understanding of the pathophysiological underpinnings of air pollution-induced diabetes is crucial to develop prevention and treatment strategies that could have an enormous public health potential.

## Results

### Gut exposure to DEP induces an inflammatory innate immune response in the gut

**Fig. 1:**
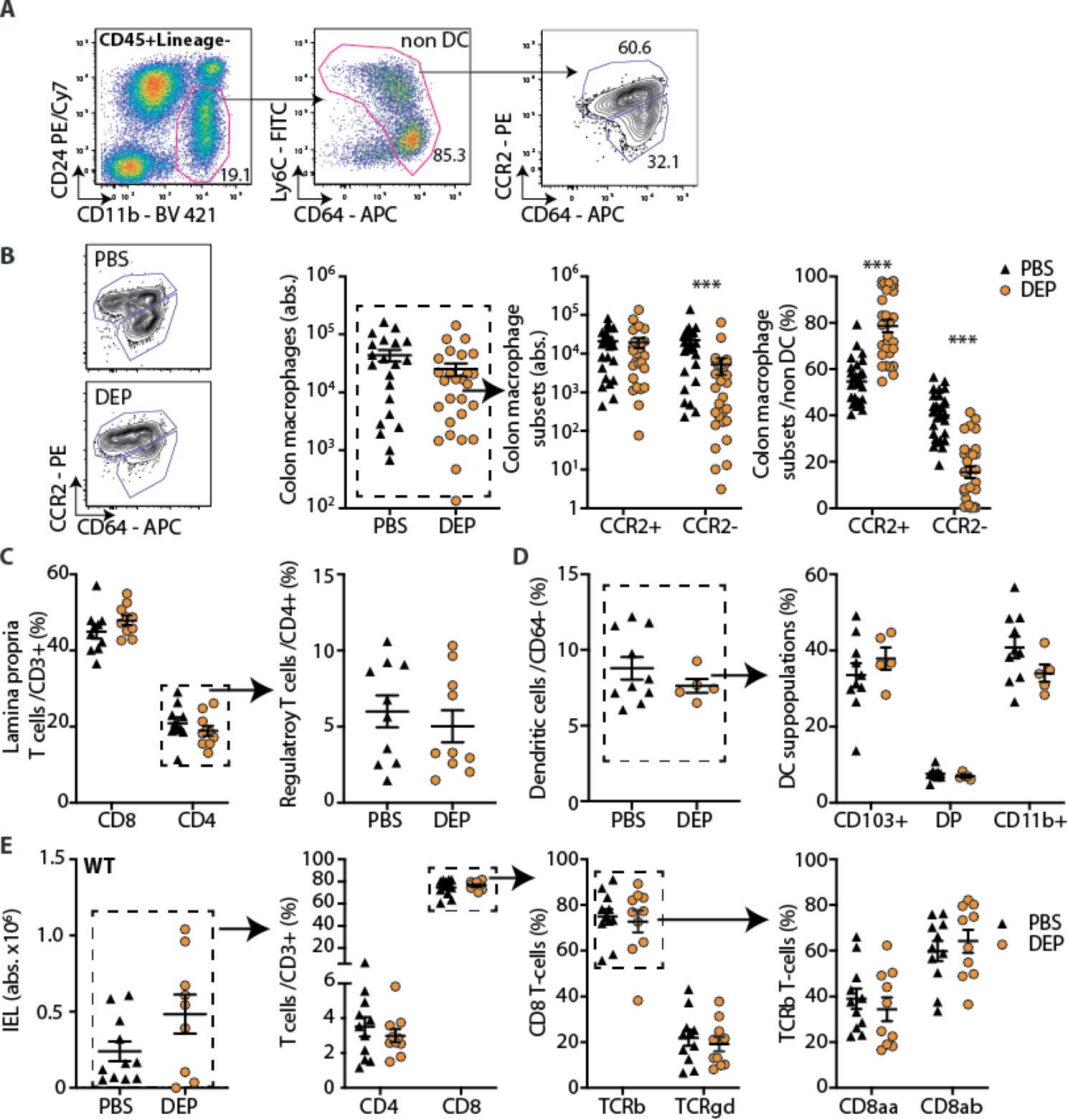
Gut exposure to DEP induces an inflammatory innate immune response in the gut. Wild-type mice were treated with 12 µg diesel exhaust particles (DEP) or phosphate-buffered saline (PBS) 5 times per week via gavage for up to 6 months. **A** Gating strategy and **B** representative flow cytometry plots, absolute numbers of total, CCR2^+^ inflammatory and CCR2^−^ anti-inflammatory/resident colon macrophages and their frequencies of total colon macrophages. **C** Frequencies of lamina propria CD4 and CD8 T-cells (of CD3^+^ cells) and regulatory T-cells (Foxp3^+^CD25^+^ of CD4^+^ T-cells). **D** Frequencies of dendritic cells (DCs) (CD64^−^MHCII^+^CD11c^+^) and their subpopulations according to CD103 and CD11b expression (DP: double positive). **E** Intraepithelial lymphocytes (IEL; CD45^+^CD3^+^) and their subtypes (of parent gate). Data are presented as mean±SEM pooled from two (C,E) or four (B) independent experiments with each data point representing an individual mouse. D depicts one representative experiment. ***p < 0.001, unpaired Mann-Whitney U test with two tailed distribution. For gating strategies see Additional File: Figure S1. DEP: Diesel exhaust particles, PBS: Phosphate-buffered saline

We previously showed that oral exposure to air pollutants resulted in an insulin secretory defect, potentially instigated by an inflammatory milieu in the gut [9]. To characterize the immune response triggered by air pollutants in the gut, we exposed mice on standard diet to diesel exhaust particles (DEP, 12 µg/day) or PBS (phosphate-buffered saline; control) by gavage and performed flow cytometry of intestinal immune cell populations (Fig. 1A, Additional File 1: Figure S1). Intestinal macrophages differentiate from recruited monocytes first into CCR2^+^ pro-inflammatory and then to CCR2^−^ anti-inflammatory/resident macrophages to maintain tissue homeostatic functions [17, 19]. Upon oral DEP exposure, we found a decrease of colonic CCR2^−^ anti-inflammatory/resident macrophages resulting in a relative increase in CCR2^+^ pro-inflammatory macrophages (Fig. 1B), indicative for an altered macrophage differentiation in the gut. As macrophage differentiation depends on the aryl hydrocarbon receptor (Ahr) [39] and the gene of its negative repressor *Ahrr* is known to be hypomethylated upon exposure to pollutants [40], we assessed DNA methylation of *Ahrr* in colon macrophages as a potential explanation for altered macrophage differentiation. However, there was no significant methylation difference at *Ahrr* in mice orally exposed to DEP (Additional File 1: Figure S2).

In contrast to the observed changes in innate immunity, lamina propria T-cells, dendritic cells and their subpopulations as well as intraepithelial lymphocytes (IEL) were unchanged upon DEP exposure (Fig. 1C-E). Thus, oral exposure to air pollutants led to an innate immune response in the gut, which was characterized by a shift of CCR2^+^ pro-inflammatory and CCR2^−^ anti-inflammatory/resident macrophage subpopulations towards an inflammatory milieu.

### DEP-induced gut inflammation and glucose intolerance do not depend on adaptive immunity

**Fig. 2:**
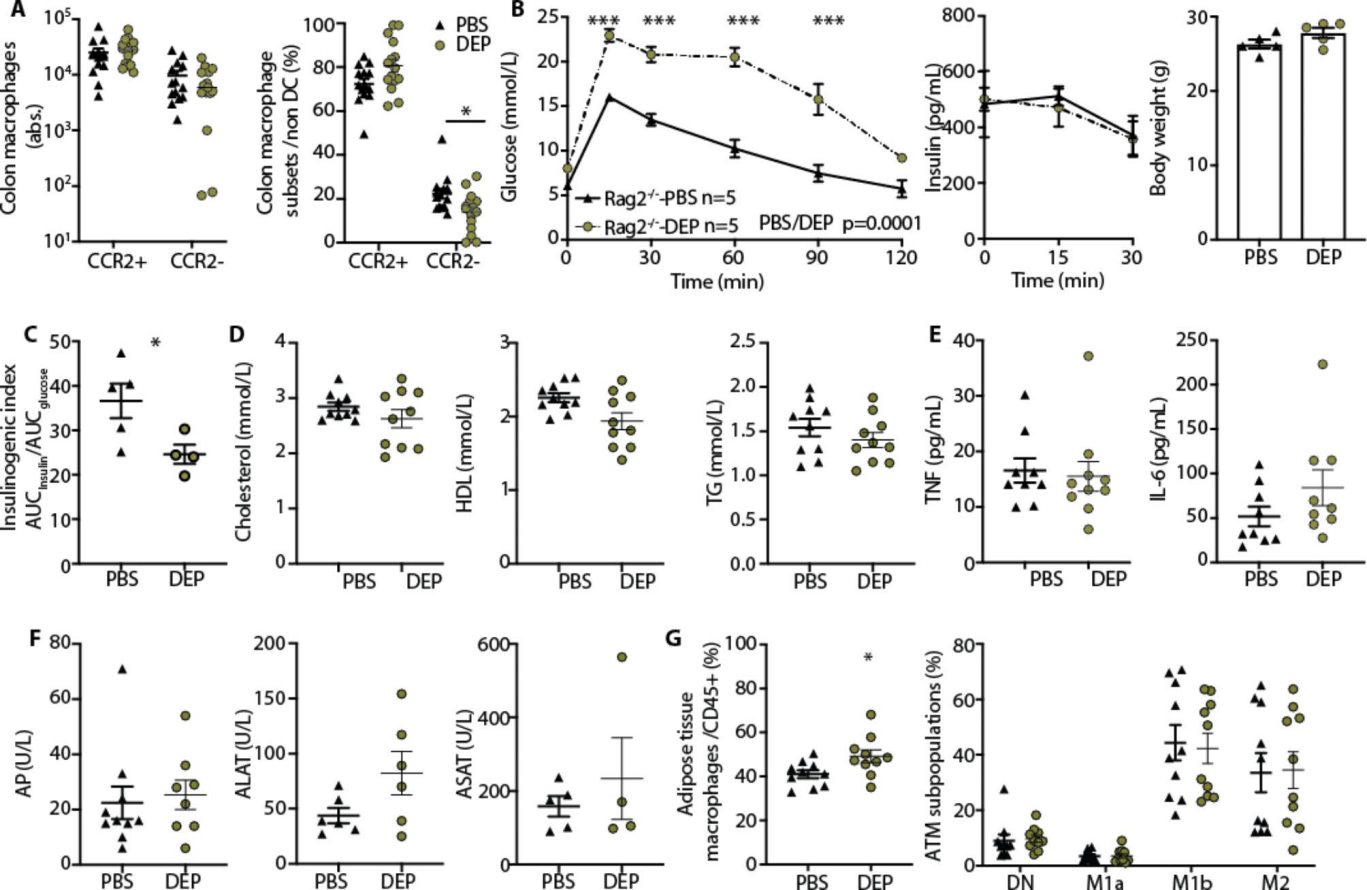
DEP-induced gut inflammation and glucose intolerance do not depend on adaptive immunity. Oral exposure of Rag2-/- mice to diesel exhaust particles (DEP) or PBS for 4 months. **A** Absolute numbers and frequencies of CCR2^+^ pro-inflammatory and CCR2^−^ anti-inflammatory/resident colon macrophages. **B** Glucose tolerance tests (GTT), insulin and body weight. **C** Insulinogenic index (ratio of AUC insulin and glucose). **D** Cholesterol, high-density lipoproteins (HDL), and triglycerides (TG). **E** Plasma TNF and IL-6. **F** Liver enzymes alkaline phosphatase (AP) and alanine transaminase (ALAT). **G** Frequencies of adipose tissue macrophages (ATM; F4/80^+^ among CD45^+^) and subpopulations DN: double negative. Data are shown as mean±SEM of pooled data from two independent experiments, with each data point representing an individual mouse. GTT and insulin values were compared by two-way ANOVA, all other parameters by a two-tailed, unpaired Mann-Whitney U test with two tailed distribution (*p < 0.05, **p < 0.01, ***p < 0.001). DEP: Diesel exhaust particles, PBS: Phosphate-buffered saline

Although we did not find changes in mucosal adaptive immune cells upon oral DEP exposure, we aimed to conclusively assess whether the adaptive immune system is required to mediate air pollution-induced glucose intolerance. To this end, we exposed Rag2-/- mice with a defective recombinant activation gene 2 (Rag2), leading to an inability in initiating V(D)J rearrangement and thereby lacking mature T- and B-lymphocytes, to DEP or PBS and characterized intestinal immune cells and glucose homeostasis. Compared to C57BL/6 N wild-type mice, Rag2-/- mice harbored a higher proportion of pro-inflammatory subpopulations irrespective of DEP exposure (C57BL/6 N mice: Fig. 1B, Rag2-/- mice: Fig. 2A), indicative of an enhanced innate immunity in these mice. Upon oral DEP exposure, Rag2-/- mice similarly exhibited a decrease in colonic CCR2^−^ anti-inflammatory/resident macrophages to what we previously observed in wild-type mice (Figs. 1B and 2A).

Rag2-/- mice developed glucose intolerance already after one month of DEP exposure compared to PBS, while insulin and body weights were comparable (Fig. 2B). The insulinogenic index was reduced in Rag2-/- mice exposed to DEP (Fig. 2C), indicative for a beta-cell defect similar to what we previously found in wild-type mice [9]. Rag2-/- mice exposed to DEP had comparable levels of systemic inflammation, lipids and liver enzymes compared to PBS treated mice (Fig. 2D-F). While adipose tissue macrophages were slightly increased in DEP exposed Rag2-/- mice, the distribution of subpopulations remained unaltered (Fig. 2G). Hence, Rag2-/- mice were not protected from air pollution-induced glucose intolerance, indicating that air pollution-induced glucose intolerance is not mediated through mature T-/B-lymphocytes.

### CCR2-/- mice with deficient monocyte recruitment are prevented from DEP-induced gut inflammation and glucose intolerance

**Fig. 3:**
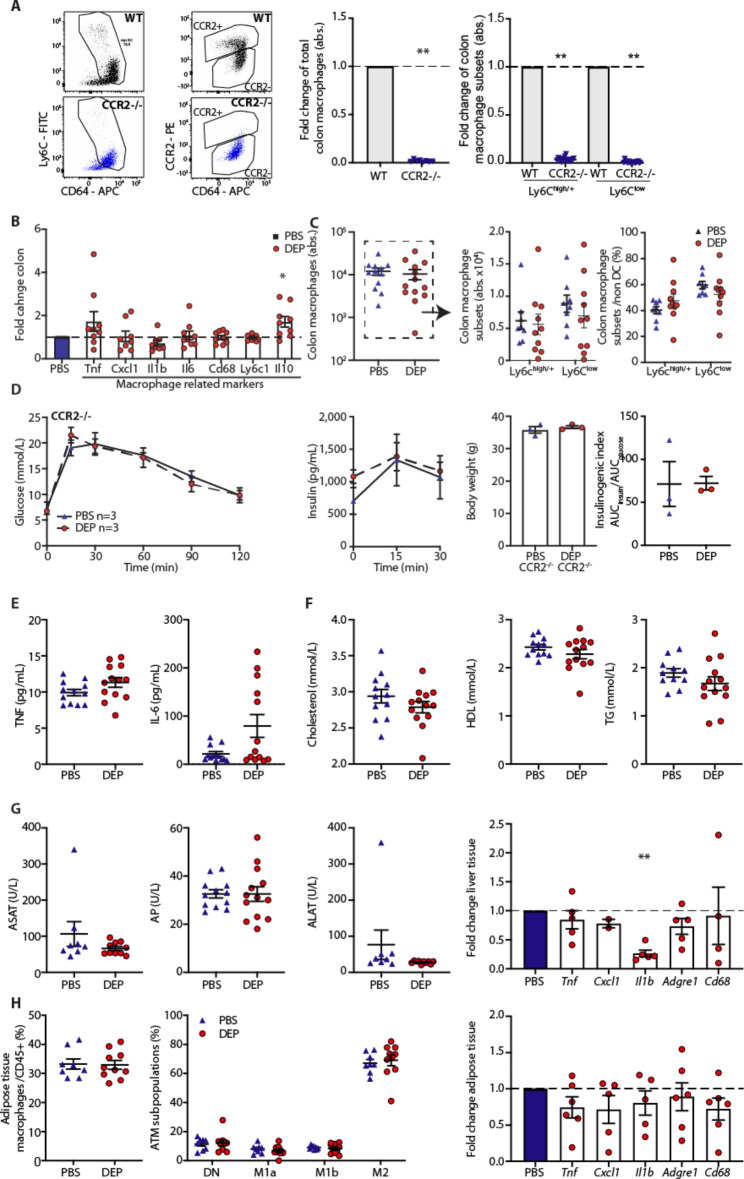
CCR2-/- mice with deficient monocyte recruitment are prevented from DEP-induced gut inflammation and glucose intolerance. CCR2-/- mice were orally exposed to diesel exhaust particles (DEP) or PBS for 8 months. **A** Representative flow cytometry plots of total colon macrophages and CCR2^+^ and CCR2^−^ macrophages. Fold change of total, Ly6C^high^ inflammatory and Ly6C^low^ anti-inflammatory/resident colon macrophages in CCR2-/- mice compared to wild-type mice. **B** Colon gene expression of immune cell markers in CCR2-/- mice exposed to DEP relative to PBS after 8 months of exposure. **C** Absolute numbers of total, Ly6C^high^ inflammatory and Ly6C^−^ anti-inflammatory/resident colon macrophages and their frequencies. **D** Glucose tolerance tests (GTT), insulin, body weight and insulinogenic index. **E** Plasma TNF and IL-6. **F** Cholesterol, high-density lipoproteins (HDL), and triglycerides (TG). **G** Liver enzymes and inflammatory gene expression in the liver. **H** Frequencies of macrophages in the adipose tissue (ATM) among CD45^+^ cells, relative distribution of ATM subpopulations, and inflammatory gene expression in adipose tissue of CCR2-/- mice exposed to DEP compared to control mice exposed to PBS. Data are presented as mean±SEM pooled from three experiments, with each data point representing an individual mouse. **p < 0.01, unpaired Mann-Whitney U test with two tailed distribution. Glucose and insulin measures were compared by two-way ANOVA. DEP: Diesel exhaust particles, PBS: Phosphate-buffered saline

Next, we addressed the role of innate immune cells in the development of DEP-induced glucose intolerance. To assess the contribution of monocyte-recruited macrophages, we exposed CCR2-/- mice to DEP or PBS by gavage. CCR2-/- mice have a bone marrow egress defect [41] and lack CCR2-dependent monocyte recruitment [36]. Independent of DEP exposure, pro-inflammatory intestinal macrophages (as defined by the expression of Ly6C) were depleted by 96.2±2.4% and anti-inflammatory macrophages by 98.8±1.4% in CCR2-/- mice (Fig. 3A). When orally exposed to DEP for 8 months, CCR2-/- mice did not develop a pro-inflammatory milieu in the gut as evidenced by unchanged gene expression of inflammatory and macrophage markers, while expression of the anti-inflammatory gene *IL-10* was elevated (Fig. 3B). For a more detailed immune cell phenotyping, we assessed macrophage subpopulations by flow cytometry in DEP und PBS exposed CCR2-/- mice. In contrast to wild-type mice, CCR2-/- mice did not exhibit an altered balance between the pro-inflammatory Ly6c^high/+^ and anti-inflammatory Ly6c^low^ intestinal macrophages upon oral DEP exposure (Fig. 3C).

Moreover, CCR2-/- mice were protected from air pollution-induced glucose intolerance and beta-cell dysfunction, suggesting a link between the level of gut inflammation instigated by DEP and glucose intolerance (Fig. 3D). Dyslipidemia, systemic, liver, and adipose tissue inflammation did not develop upon DEP exposure, similar to our previous findings in wild-type mice (Fig. 3E-H). Thus, CCR2-/- mice were protected from gut inflammation and glucose intolerance by oral exposure to DEP.

### Mice treated with the CSF1R-inhibitor PLX5622 are devoid of tissue resident macrophages and protected from DEP-induced gut inflammation and glucose intolerance

**Fig. 4:**
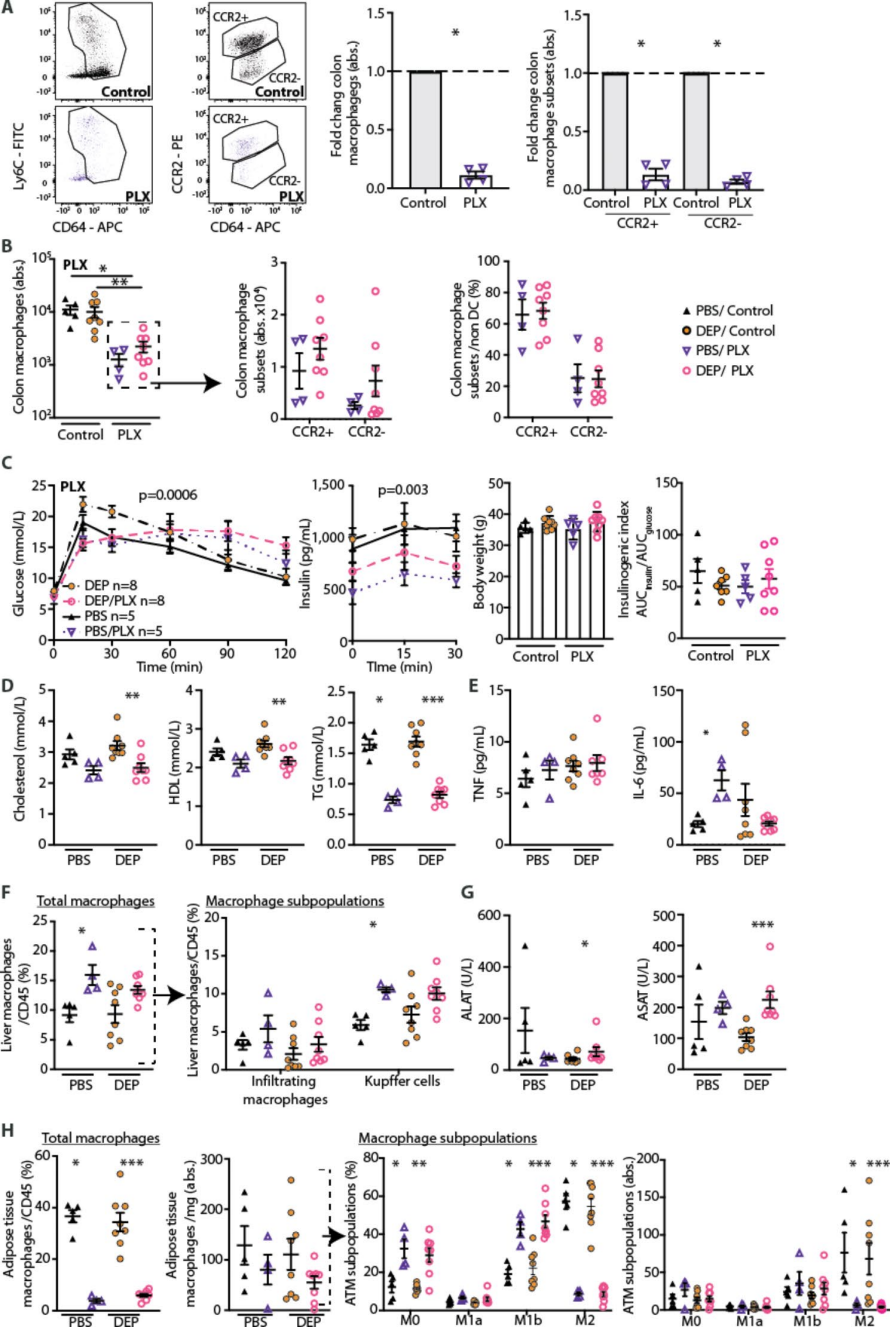
Mice treated with the CSF1R-inhibitor PLX5622 are devoid of tissue resident macrophages and protected from DEP-induced gut inflammation and glucose intolerance. Wild-type were mice pharmacologically depleted of macrophages by a diet containing the CSF1R-inhibitor PLX5622 and exposed to diesel exhaust particles (DEP) or PBS for 10 months. **A** Representative flow cytometry plots of colon macrophages and their subsets and fold change of absolute numbers in mice treated with PLX5622 compared to untreated controls. **B** Absolute numbers of total, CCR2^+^ inflammatory and CCR2^−^ anti-inflammatory/resident colon macrophages and their frequencies. **C** GTT, insulin, body weight and insulinogenic index. **D** Cholesterol, high-density lipoproteins (HDL), and triglycerides (TG). **E** Plasma TNF and IL-6. **F** Flow cytometric analysis of liver macrophages of CD45^+^ cells. CD11b was used to distinguish infiltrating macrophages (CD11b^high^) and Kupffer cells (CD11b^low^). **G** Liver enzymes. **H** Frequencies of adipose tissue macrophages (ATM) among CD45^+^ cells, distribution of ATM subpopulations among ATM. Data are presented as mean±SEM pooled from two independent experiments with each data point representing an individual mouse. *p < 0.05, **p < 0.01, ***p < 0.001, unpaired Mann-Whitney U test with two tailed distribution. Insulin and glucose values were analyzed using two-way ANOVA. DEP: Diesel exhaust particles, PBS: Phosphate-buffered saline, PLX: PLX5622.

As a pharmacological approach to deplete tissue resident macrophages, we used the Colony stimulating factor 1 receptor (CSF1R)-inhibitor PLX5622 and exposed these mice orally to DEP or PBS. CSF1R is considered as a typical macrophage maintenance factor [42]. Independent of DEP exposure, CCR2^+^ pro-inflammatory and CCR2^−^ anti-inflammatory/resident intestinal macrophages were strongly reduced in mice treated with PLX5622 (86.7±9.9% and 92.8±3.8%, respectively) (Fig. 4A). Upon DEP exposure for 10 months, the proportions of CCR2^+^ pro-inflammatory and CCR2^−^ anti-inflammatory/resident macrophage subpopulations in the gut were comparable between CSF1R- and PBS-treated mice, indicative for an absent innate immune response (Fig. 4B).

Irrespective of DEP exposure, mice treated with the CSF1R-inhibitor PLX5622 had an improved glucose tolerance at the 15 min timepoint, which was impaired after time point 30 min compared to controls, while insulin levels were reduced at all timepoints (Fig. 4C). Upon DEP exposure, glucose tolerance, insulin levels, body weights and the insulinogenic index as a measure for beta-cell function were comparable between PLX5622 treated mice and controls (Fig. 4C). While already CSF1R-inhibition by PLX5622 decreased systemic lipid levels and increased liver enzymes, DEP did not have an additional effect on lipids, systemic inflammation markers, liver macrophages and enzymes, and adipose tissue inflammation in PLX5622-treated mice (Fig. 4D-H). In sum, pharmacological macrophage depletion by the CSF1R-inhibitor PLX5622 protected mice from DEP-induced gut inflammation and glucose intolerance.

### The transcriptional response upon DEP involves inflammatory and interferon responses in colon macrophages

**Fig. 5:**
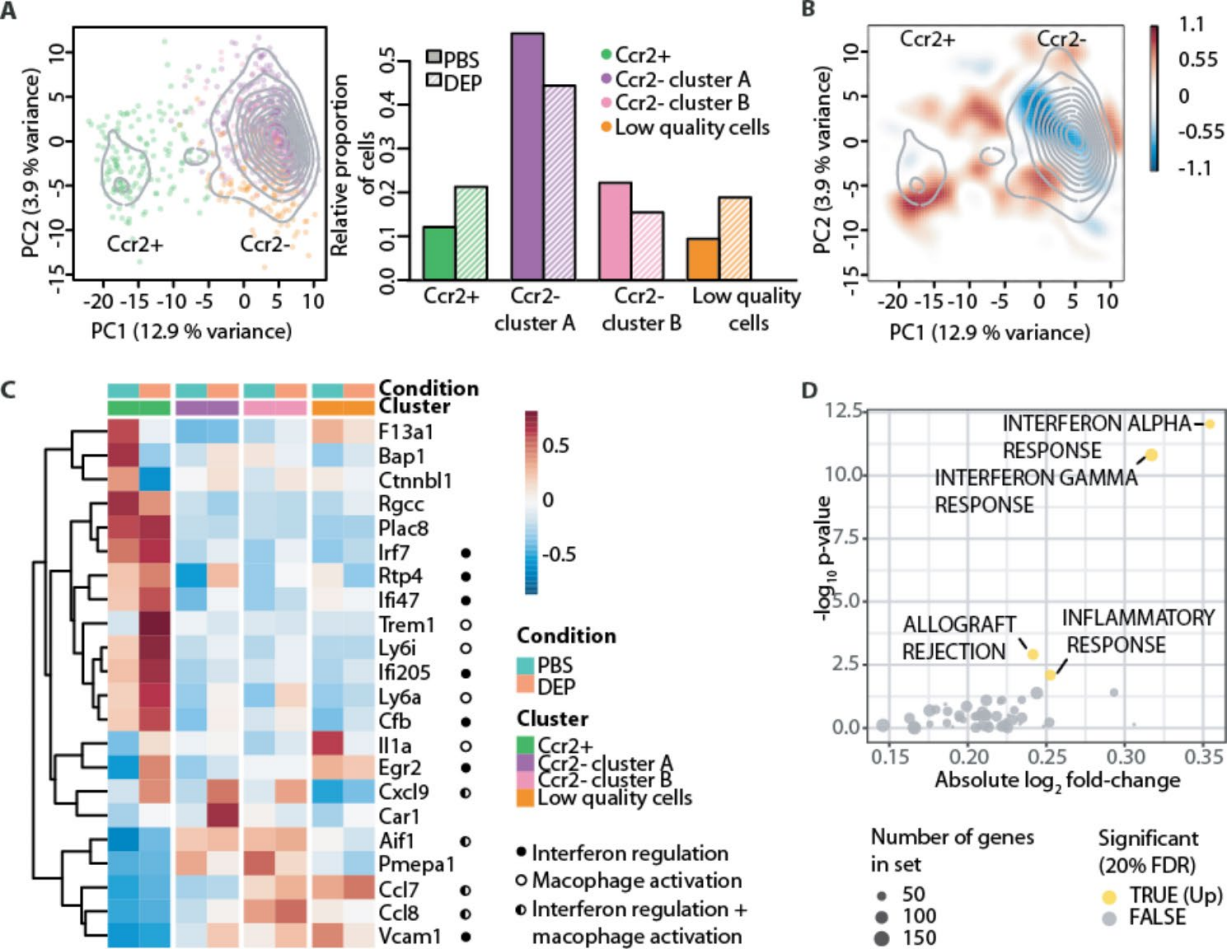
The transcriptional response upon DEP involves inflammatory and interferon responses in colon macrophages. **A** Principal component analysis of colon macrophages from diesel exhaust particles (DEP) or PBS-exposed mice, showing *Ccr2*^+^ and *Ccr2*^−^ subpopulations (colors represent clusters; contours cell density) and relative proportion across clusters. **B** Differential 2D density plot (red indicates excess of DEP-, blue excess of PBS-exposed cells). **C** Cluster-specific differential gene expression (closed circles indicate genes related to interferon regulation, open circles genes related to macrophage activation, half-open circles to both). **D** Significantly up-regulated MSigDB Hallmark pathways upon DEP exposure. One experiment with n = 2. DEP: Diesel exhaust particles, FDR: false discovery rate, PBS: Phosphate-buffered saline

To interrogate potential underlying signaling pathways, which might give reference to the crosstalk between colonic macrophages and β-cells, we performed single cell RNA-sequencing (RNA-seq) of colon macrophages of wild-type mice orally exposed to DEP or PBS. By using unbiased hierarchical clustering of cells, Ccr2^+^ pro-inflammatory and Ccr2^−^ anti-inflammatory/resident macrophages were identified as the two main populations (the latter comprising two related clusters, Fig. 5A). First, we corroborated the increase in abundance of Ccr2^+^ relative to Ccr2^−^ macrophages upon DEP exposure (Fig. 5A,B). Subsequently, we compared the transcriptomes stratifying the analysis by cell clusters to correct for differential abundance. Many genes, which were differentially expressed between DEP and PBS exposed mice, concerned macrophage activation, interferon regulation or both (Fig. 5C; Additional File Figure S3). Accordingly, gene set enrichment analysis (GSEA) using MSigDB Hallmark pathways showed up-regulation of inflammatory, interferon α and γ responses, as well as allograft rejection in DEP exposed mice (Fig. 5D). Hence, the transcriptional response upon oral DEP exposure corroborated an inflammatory shift in intestinal macrophage subpopulations and uncovered that the transcriptional response involved inflammatory and interferon responses.

### NLRP3 inflammasome activation contributes to DEP-induced gut inflammation and glucose intolerance

**Fig. 6:**
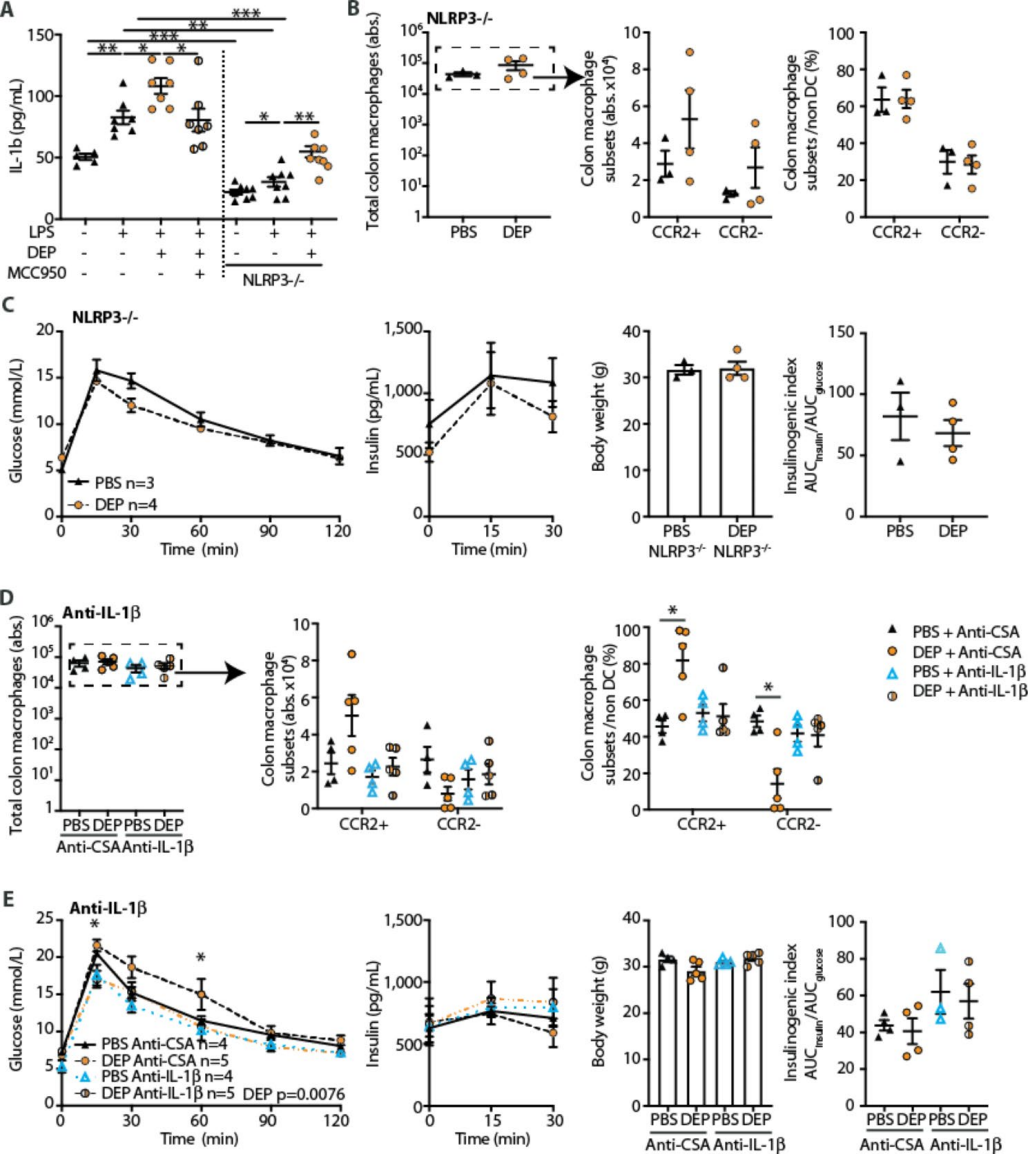
NLRP3 inflammasome activation and IL-1β secretion contribute to DEP-induced gut inflammation and glucose intolerance. **A** Protein expression of IL-1β in supernatant of wild-type and Nlrp3-/- peritoneal macrophages exposed in vitro to 125 µg/mL diesel exhaust particles (DEP) or PBS in the presence or absence of MCC950 (Nlrp3-inhibitor). **B** Absolute numbers of total, CCR2^+^ pro-inflammatory and CCR2^−^ anti-inflammatory/resident colon macrophages and their frequencies in Nlrp3-/- mice exposed to diesel exhaust particles (DEP) or PBS for 6 months. **C** GTT, insulin, body weight and insulinogenic index. **D-E** Wild-type mice were treated with anti-IL-1β or control antibody (anti-cyclosporin A; CSA) after they developed impaired glucose tolerance. **D** Absolute numbers of total, CCR2^+^ pro-inflammatory and CCR2^−^ anti-inflammatory/resident colon macrophages. **E** GTT, insulin, body weight and insulinogenic index after two weeks of anti-IL-1β treatment. Data are shown as mean±SEM. B-E represent one experiment, A pooled data from two independent experiments. *p < 0.05, **p < 0.01, ***p < 0.001, unpaired Mann-Whitney U test with two tailed distribution, two-way ANOVA was used to analyze insulin and glucose values. DEP: Diesel exhaust particles, LPS: Lipopolysaccharide, PBS: Phosphate-buffered saline

Hence, the transcriptional pattern indicated inflammatory processes to be involved in intestinal macrophages exposed to DEP. Because of its prominent role in orchestrating immune tolerance and driving inflammatory responses to pathogens in the gut [43], the NOD-, LRR- and pyrin domain-containing protein 3 (NLRP3) inflammasome represents a potential candidate for mounting an innate immune response upon sensing air pollutants reaching the gastrointestinal tract. Upon activation, NLRP3 is known to recruit apoptosis associated speck like protein (ASC) and activate serine protease caspase-1 (Pro-caspase-1) to form caspase-1, which is a prerequisite for the cleavage and maturation of the inflammatory cytokines interleukin-1β (IL-1β) and interleukin-18 (IL-18) [44]. We hypothesized that NLRP3 could be involved in air pollution-induced gut inflammation and glucose intolerance. To address this, we first used an in vitro approach. We treated peritoneal macrophages that were primed by LPS with DEP or PBS and measured NLRP3 activation by IL-1β secretion. Activation with DEP significantly upregulated the secretion of IL-1β in LPS-primed peritoneal macrophages (Fig. 6A). Additional treatment with the NLRP3-inhibitor MCC950 prevented the DEP-induced increase in IL-1β secretion (Fig. 6A). Peritoneal macrophages from Nlrp3-/- mice exhibited a similar pattern of IL-1β compared to wild-type mice, however, at 3-fold lower levels (Fig. 6A).

Next, to test the role of NLRP3 on gut inflammation and glucose tolerance in vivo, we exposed Nlrp3-/- mice to DEP or PBS for 6 months. When we compared macrophage subpopulations between DEP und PBS exposed Nlrp3-/- mice, the proportion of CCR2^+^ pro-inflammatory and CCR2^−^ anti-inflammatory/resident macrophage subpopulations in the gut were not changed upon DEP exposure (Fig. 6B). Regarding the glycemic control upon DEP exposure, Nlrp3-/- mice did not develop glucose intolerance. Also, insulin levels, body weights and the insulinogenic index were similar in DEP compared to PBS exposed Nlrp3-/- mice (Fig. 6C). In summary, DEP contributes to NRLP3 activation and IL-1β secretion in vitro. Conversely, genetic depletion of the NLRP3 inflammasome in mice prevented DEP-induced gut inflammation and glucose intolerance.

### DEP-induced gut inflammation and glucose intolerance are reversed by pharmacological inhibition of IL-1β

Lastly, we assessed whether the involvement of NLRP3 in air pollution-induced glucose intolerance was mediated by the secretion of IL-1β as a potential target for a therapeutic intervention. We exposed wild-type mice to DEP or PBS and treated these mice with a monoclonal antibody against IL-1β or CSA control antibody after they developed impaired glucose tolerance as assessed by monthly glucose tolerance tests. As previously observed, DEP led to a decrease in colonic CCR2^−^ anti-inflammatory/resident macrophages in mice treated with the CSA control antibody, resulting in a relative increase in CCR2^+^ pro-inflammatory macrophages (Fig. 6D). In mice treated with the IL-1β antibody, however, the balance between CCR2^+^ pro-inflammatory and CCR2^−^ anti-inflammatory/resident macrophage subpopulations was restored (Fig. 6D). Glucose tolerance upon DEP exposure was impaired in mice treated with the CSA control antibody, but reverted back to normoglycemia after two weeks of IL-1β antibody treatment despite ongoing DEP exposure (Fig. 6E). Taken together, DEP-induced gut inflammation and glucose intolerance were reversed by pharmacological inhibition of IL-1β.

## Discussion

**Fig. 7:**
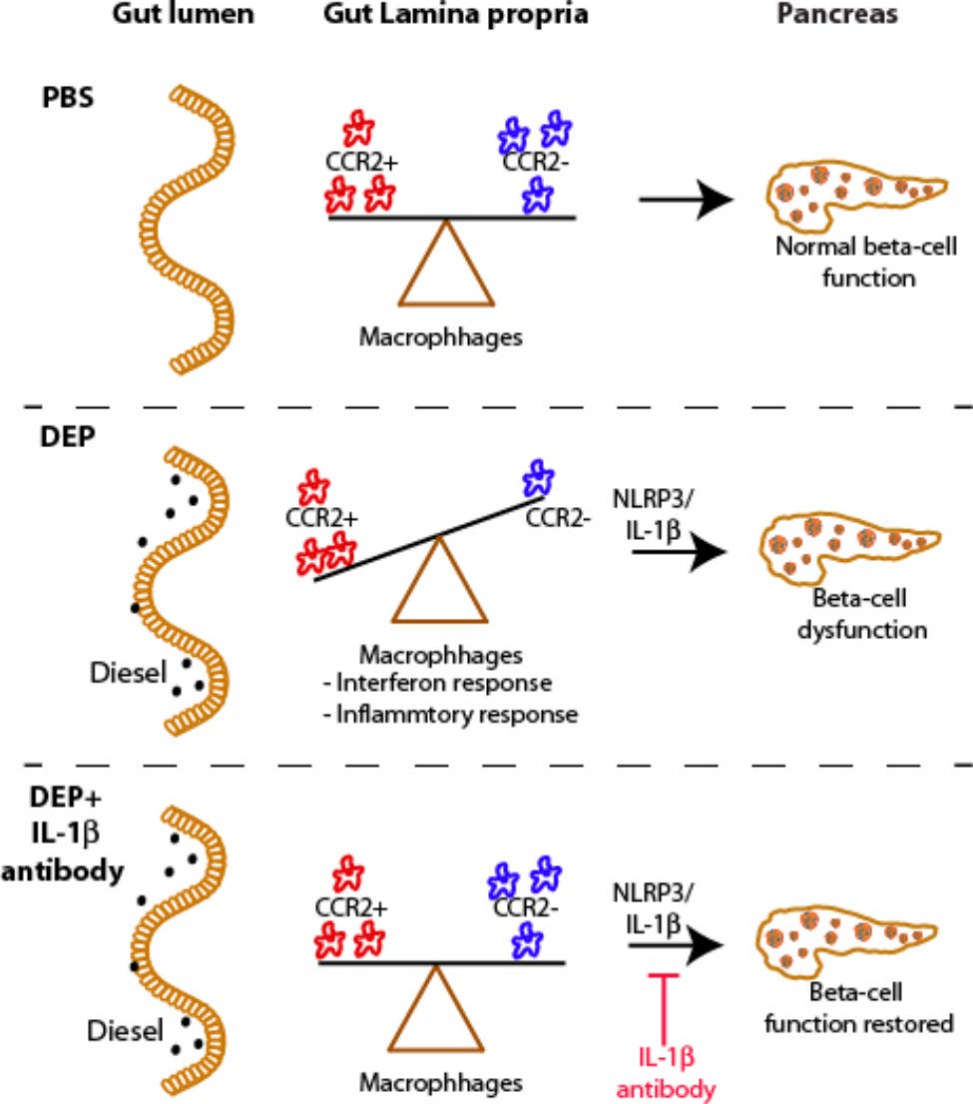
Graphical abstract. Gut exposure to diesel exhaust particles (DEP) shifts intestinal macrophages to an inflammatory state and induces beta-cell dysfunction, which can be restored by anti-IL-1β treatment. DEP: Diesel exhaust particles, PBS: Phosphate-buffered saline

Our results show that oral exposure to DEP leads to a shift of intestinal macrophage subpopulations towards an inflammatory milieu in the gut (Fig. 7). Intestinal macrophages in the gastrointestinal tract have a specific differentiation trajectory, arising from extravasated monocytes that differentiate through a CCR2^+^ pro-inflammatory stage to become CCR2^−^ anti-inflammatory/resident macrophages with tissue homeostatic functions [16]. These distinct intestinal macrophage subpopulations have to be taken into account by appropriate gating strategies [17, 19]. In contrast to inflammatory bowel disease (IBD) or infectious diseases of the gut, the inflammatory milieu instigated by DEP is not a consequence of increased CCR2^+^ pro-inflammatory macrophages, but decreased CCR2^−^ anti-inflammatory/resident macrophages. This finding speaks against enhanced monocyte recruitment as an underlying mechanism of DEP-induced gut inflammation. CCR2^−^ anti-inflammatory/resident macrophages could be reduced due to phagocytosis-induced cell death, however, markers of cell death were not increased in our RNA-Seq data. It seems more likely that the altered transcriptional program upon DEP leads to disrupted intestinal macrophage differentiation. Hence, while our data show an inflammatory milieu due to a loss in CCR2^−^ anti-inflammatory/resident macrophages, the fate of the “lost” CCR2^−^ anti-inflammatory/resident subset needs be addressed in future studies.

To link the DEP-induced changes in mucosal immunity to glucose metabolism, we used mouse models with either defective adaptive or innate immunity and exposed them to DEP or PBS. These mouse models have previously not shown any metabolic traits – i.e., glucose intolerance or insulin resistance – on a chow diet (CCR2-/- [45–47] and Rag2-/- mice [48]). Short-term treatment with CSF1R-inhibitors also did not affect glucose homeostasis in chow fed lean mice [49, 50]. However, long-term CSF1R-inhibition by PLX5622 in our study yielded a slightly altered curve of glucose tolerance together with reduced insulin levels, which was independent of DEP exposure.

Rag2-/- mice were not protected from air pollution-induced glucose intolerance. In contrast to wild-type mice, who require several months of air pollution exposure to develop a metabolic phenotype [9], Rag2-/- mice developed glucose intolerance after just one month of DEP administrations. This could be potentially linked to the heightened innate immune response in the gastrointestinal tract as shown by higher proportions of CCR2^+^ intestinal macrophages compared to wild-type mice. Such a heightened innate immune response in Rag2-/- mice is consistent with previous literature and has been explained by a lack of lymphocytes suppressing innate immune cell function [51]. On the contrary, mouse models lacking major components of innate immunity by either genetic (CCR2-/- mice) or pharmacological manipulation (CSF1R-inhibition) were protected from DEP-induced gut inflammation and glucose intolerance. Thus, mouse models defective of adaptive or innate immunity revealed that macrophages are involved in mediating air pollution-induced induced glucose intolerance upon gut exposure to DEP.

The transcriptional response of mice exposed to DEP involved an inflammatory and interferon response in colonic macrophages. Because of its prominent role in immune tolerance and inflammatory responses to pathogens in the gut [43], we tested NLRP3 as a potential candidate for the innate immune response upon DEP exposure. NLRP3 activation has been described in response to hyperglycemia, saturated fatty acids, and islet amyloid polypeptide and is linked to glucose intolerance, insulin resistance, and beta-cell death [52–56]. Further evidence suggestive for a link between the NLRP3/IL-1β axis and glucose metabolism comes from studies that use anti-inflammatory approaches such as IL-1β inhibition to improve glycemic control [57]. Particulate matter has also been described to activate the NLRP3 inflammasome [58], but previous studies mainly focused on lung and brain tissue [59, 60]. We confirmed by an in vitro approach that IL-1β secretion was indeed enhanced upon DEP and dampened by pharmacological or genetic NLRP3 depletion. However, other inflammasomes besides NLRP3 could also be involved [61] as macrophages of Nlrp3-/- mice showed a measurable increase in IL-1β upon DEP exposure. The DEP-induced IL-1β secretion in Nlrp3-/- mice was equivalent to baseline levels of wild-type mice, suggesting that the NLRP3 inflammasome activation by DEP in wild-type mice exceeds a critical threshold to induce pro-inflammatory macrophages or perturb their differentiation. The role of NLRP3 signaling was also confirmed in vivo as deletion of NLRP3 or anti-IL-1β treatment reversed the pro-inflammatory milieu in the gut, insulin secretion defect and glucose intolerance. These findings point towards therapeutic strategies, encompassing pharmacological manipulation of the NLRP3/IL-1β axis.

There are several open questions and limitations that need to be addressed in future studies: First, we cannot draw conclusions regarding the contribution of lung exposure to the metabolic dysfunction as the particles were exclusively introduced into the gut. It is plausible that both gut and lung exposure play a collective role in shaping the metabolic phenotype, potentially through distinct disease mechanisms. For example, lung exposure leads to hypercholesterinemia and increased liver lipids, which could eventually lead to insulin resistance once the accumulation of liver lipids surpasses a certain threshold [9]. On the other hand, particulate (ingested) and gaseous (inhaled) components of air pollution might engage different disease mechanisms, as evidenced by acute impacts on glucose metabolism from ozone exposure, while air pollutant particles administered via the gastrointestinal tract take several months to induce a metabolic phenotype. Second, it is not known what cues intestinal macrophages respond to, i.e., air pollution particles, changes in gut microbiota, or metabolites. Previous studies demonstrated changes in gut microbiota upon inhalational [62–64] and oral air pollution exposures [65–67]. As there are close interactions between microbiota and intestinal immune cells [68], altered microbiota or their metabolites could contribute to the changes in intestinal macrophages we observed. Third, the question remains how altered intestinal macrophages cross-talk with pancreatic islets. For example, colon macrophages could traffic from the gut to pancreatic islets or secreted factors such as the cytokine IL-1β could disseminate to the pancreas via the blood circulation, lymphatic vessels or neuronal circuits. Besides the sympathetic and parasympathetic nervous systems, enteric-associated neurons have been shown to directly link the gut and pancreas and thereby impact on metabolic control [69]. Fourth, other pollutants such as particulate matter or persistent organic pollutants have also been linked with an increased diabetes risk [7, 8, 70]. Whether these pollutants mediate the increased diabetes risk via similar immune-mediated pathways in the gut as DEP needs to be addressed in future studies.

Taken together, our study proposes a role of gut innate immunity in air pollution-induced diabetes. The proposed role of an inflammatory innate immune response by decrease in CCR2^−^ anti-inflammatory/resident macrophages in the gut adds a new dimension to the interface between environmental pollutants and metabolic health. Therapeutic inhibition of the NLRP3/IL-1β system protected mice from air pollution-induced diabetes and thus points towards potential therapeutic strategies. Given the global burden of diabetes and the ever-increasing plight of air pollution in numerous regions across the world, these findings are of high clinical significance towards building a healthier society.

## Methods

### Study design

To study the mechanisms underlying air pollution-induced glucose intolerance upon gavage with DEP, we used unbiased RNA-seq as well as Rag2-/-, CCR2-/- and Nlrp3-/- mice and pharmacological depletion of IL-1β or macrophages by CSF1R-inhibition. All experiments were carried out two to four times, except for the experiments with the neutralizing antibody, NLRP3-/- mice and RNA-sequencing, which were carried out once. The mice were rendered into groups by weight matching. No further randomization was performed. Blinding was not feasible during the treatment of the mice. However, results were analyzed in a blinded fashion whenever possible. Group and sample sizes for each experiment are indicated in the figure legends.

### Mice

Male C57BL/6 N mice were purchased from Charles River Laboratories and bred in house. Male CCR2-/-, Rag2-/-, and Nlrp3-/- mice were bred in our facility. Mice were maintained in specific pathogen-free conditions with free access to pelleted food and water. All animal procedures were approved by the local Animal Care and Use Committee and performed in accordance with Swiss Federal regulations.

### Exposure protocol

As diesel exhaust particles (DEP), the standard reference material NIST 1650b was used, which was generated from four-cycle engines after 200 operating hours, representing heavy-duty diesel engine. DEP were dissolved in sterile PBS, sonicated for 2 h to ensure homogeneity and stored as aliquots at room temperature. The suspension was vortexed directly before use and delivered to the gut by gavage in a volume of 200 µL for up to 10 months with a daily dose of 12 µg/day (5 days a week), while the control group received PBS. The weekly dose added up to 60 µg DEP corresponding to an average daily dose of 8.6 µg/mouse which is approximately equivalent to a daily inhalation exposure of 160 µg/m^3^ (calculated by the daily exposure divided by the daily inhaled air volume). For the dose calculation see reference [9].

### Pharmacological depletion of macrophages

Macrophages were depleted by applying a diet containing the CSF1R-inhibitor PLX5622 (1200 ppm), or control diet, from the age of 4–5 weeks onwards (Cat# D10001i, Research Diets).

### Glucose tolerance tests (GTTs)

After 6 h of fasting, mice received a glucose bolus intraperitoneally (2 g/kg body weight, Braun). Blood glucose was measured at 0, 15, 30, 60, 90 and 120 min and blood was collected at time points 0, 15 and 30 min for insulin measurements.

### Isolation and flow cytometric assessment of immune cells

Cells of the colon and adipose tissue were isolated by enzymatic digestion as follows:

The colon was washed in HBSS (Cat# 14,185,052, Gibco), cut into small pieces, placed on an orbital shaker at 37 °C, and washed twice 10 min in HBSS containing 2 mM EDTA (Cat# 03690, Sigma), rinsed twice with HBSS prior to digestion with 1 mg/mL collagenase VIII (Cat# C2139, Sigma-Aldrich) and 12.5 µg/mL DNase I (Cat# 11,284,932,001, Roche), 30 min. shaking at 37 °C, followed by homogenization with a gentleMACS Dissociator (Cat# 130-096-427, Milteny Biotec) using the mouse intestine program. Cells of the lamina propria were enriched for leukocytes using a Percoll gradient (Cat# GE17-0891-01, GE Healthcare; 40% and 70%). IEL were isolated from colon tissue by two rounds of 4 min vortexing, prior to digestion, with maximal speed and enriched using Percoll. Single cell suspensions were stained for 30 min. at 4 °C. Although originally described for skin macrophages, the same gating strategy can be applied to characterize colon macrophages [16, 71]. Gating see Fig. 1A and Additional File: Figure S1.

Epididymal adipose tissue was minced and digested with 1.5 mg/mL collagenase IV (Cat# LS004189, Worthington) and 8.25 µg/mL DNase I 25 min on a thermomixer at 400 rpm. Erythrocytes were removed by red cell lysis buffer. Adipose tissue macrophages were gated as CD45^+^F4/80^+^CD11b^+^ and subdivided into subpopulations double negative (DN), M1a (CD11c^+^CD206^−^), M1b (CD11c^+^CD206^int^), and M2 (CD11c^− to low^CD206^+;^ gating see [72]).

From Biolegend, we obtained antibodies against CD11b (M1/70), CD11c (N418), MHCII (M5/114.14.2), Ly6C (HK1.4), CD45 (30-F11), F4/80 (BM8), CD103 (2E7), CD24 (M1/69), CD64 (X54-5/7.1), CD3 (145-2C11), CD19 (6D5), NK1.1 (PL136), Ly6G (1A8), CD206 (C068C2). mAb for CCR2 (475,301) was purchased from R&D. mAb for Siglec F (E50-2440) was obtained from BD. For details see Additional File 1: Table S1. Cell analysis and sorting was performed on a FACS LSRII Fortessa and FACS Aria III, respectively (BD Biosciences). Acquired data were analyzed using FlowJo software (Version 9.9 or higher), TreeStar Inc. Ashland, OR, USA).

### Bisulfite pyrosequencing

Bisulfite converted DNA was used to measure methylation levels by pyrosequencing as described previously [73]. Additional File: Table S3 for primer sequences.

### Protein expression analysis

Plasma insulin, TNF, IL-1β and IL-6 were quantified by electrochemiluminescence (MESO SECTOR S 600) using kits from MesoScale Diagnostics (Cat# K152BZC, K150JWC and K15048, respectively).

### Liver enzymes and lipids

Liver enzymes and blood lipids were measured in plasma on the c502/c702 modules of the Cobas 8000 series (Roche Diagnostics).

### Gene expression analysis

RNA isolation was performed using the NucleoSpin RNA (Cat# 740,955, Macherey Nagel) or the RNeasy Plus Universal Mini kit (Cat# 73,404, QIAGEN). Reverse transcription was performed with GoScript™ (Cat# A5003, Promega). GoTaq qPCR Master Mix (Cat# A4472919, Promega) was used for real-time PCR (ViiA7, Thermo Fisher Scientific). Primer sequences (Microsynth) are listed in Additional File: Table S2.

### Single-cell RNA-sequencing

Lamina propria cells positive for CD11b and positive either for Ly6C, CD64, or both, were sorted in parallel in a single batch from 2 controls and 2 mice exposed to DEP. Cell suspensions were loaded on the wells of a single 10× Genomics Chromium Single Cell Controller (one well per mouse replicate). Single-cell capture and cDNA and library preparation were performed with a Single Cell 3’ v2 Reagent Kit (10× Genomics) according to the manufacturer’s instructions.

Quality control of the libraries produced was done by a fragment analyzer system utilizing capillary electrophoresis. Afterwards, sequencing was performed on one flow-cell of an Illumina NexSeq 500 machine at the ETH Zurich Genomics Facility in Basel. In total, 353,174,232 reads were sequenced, ranging from 67,486,133 to 108,416,151 per sample. Data were analyzed by the Bioinformatics Core Facility, Department of Biomedicine, University of Basel. Paired-end reads quality was assessed with the FastQC tool (version 0.11.5). Sequencing files were processed with the Cell Ranger software (version 3.0.2, provided by 10× Genomics at https://support.10xgenomics.com/single-cell-gene-expression/software/downloads/3.0 to perform sample and cell demultiplexing, read alignment to the mouse mm10 genome assembly with STAR, and to generate read count table. Default settings and parameters were used, except for the version of STAR updated to 2.6.1a, and the STAR parameters *outSAMmultNmax* set to 1 and *alignIntronMax* set to 10,000. The reference transcriptome “refdata-cellranger-mm10-3.0.0”, provided by 10× Genomics and based on Ensembl release 93, was used (available at http://cf.10xgenomics.com/supp/cell-exp/refdata-cellranger-mm10-3.0.0.tar.gz). The fraction of reads that mapped to the genome ranged between 80.4% and 87% per sample, and Cell Ranger reports indicated sequencing saturations (fraction of reads originating from an already-observed UMI) ranging from 95.6 to 96.9% per sample.

Samples were merged with the “cellranger aggregate” procedure without downsampling. Further analysis was performed starting from the UMI counts matrix by using the dropletUtils (version 1.5.4), scran (version 1.12), and scater (version 1.12) Bioconductor packages, following most steps illustrated in the simpleSingleCell Bioconductor workflow [74].

Based on the clearly bimodal distributions observed across cells, cells were filtered out if they had log_10_ library sizes less than 3 (i.e., a minimum of 1,000 UMIs per library), log_10_ total number of features detected less than 2.5 (i.e. a minimum of 317 genes detected), or if they had 0% (to exclude single nuclei droplets) or more than 7% (to exclude damaged cell droplets) of UMI counts attributed to the mitochondrial genes [75]. Low-abundance genes with average log_2_ counts per million reads lower than 0.002 were filtered out. A doublet detection approach involving *in silico* simulation of doublets from the single-cell expression profiles, as implemented in the scran Bioconductor package, revealed no suspicious cells. The resulting filtered dataset included expression values for 12,182 genes for 943 cells, ranging from 189 to 334 cells per sample, for a total of 420 PBS cells, and 523 DEP cells. An average of 4,497 UMIs was counted per cell, for an average of 1,336 genes was detected per cell.

The expression values (log-transformed UMI counts) were normalized with library size factors estimated from pools of cells determined based on rank correlations cross expression profiles (scran function *quickCluster*). The technical noise was assumed to follow a Poisson distribution, and a mean-variance trend was fitted to the data (*makeTechTrend* function of the scran package with default parameters). This trend was subtracted to the variance of each gene to obtain the residual “biological” component of the variance. After performing a principal component analysis (PCA) on the top 500 most variable genes, the *denoisePCA* function of the scran package was used to choose the number of dimensions to retain in order to denoise the expression matrix.

Clustering cells into putative subpopulations was done on normalized and denoised log-counts values by using hierarchical clustering on the Euclidean distances between cells (with Ward’s criterion to minimize the total variance within each cluster; package cluster version 2.0.9). The cell clusters were identified by applying a dynamic tree cut (package dynamicTreeCut, version 1.63-1), which resulted in 4 clusters. The R package SingleR was used for reference-based annotation of the cell type of cells in our dataset. We used the Immunological Genome Project (ImmGen) mouse database as reference, and we filtered out the 31 cells (out of a grand total of 943 high quality cells) not annotated as “Monocytes” or “Macrophages” as these likely represented contaminants.

Differential expression between DEP cells and PBS cells stratified by differentiation stage was performed by using a pseudo-bulk approach. The UMI counts of cells from each sample and each cluster were aggregated. Cells from cluster 4 were ignored because they most likely represent damaged or low-quality cells (i.e., they displayed no expression of specific marker genes and have a higher fraction of reads coming from mitochondrial genes). Additionally, an “overall” analysis was performed where UMI counts of all cells from each sample were pooled. The resulting 16 pseudo-bulk samples were then treated as bulk RNA-seq samples for differential expression analysis. Genes were filtered to keep those with counts per million reads sequenced values higher than 1 in at least 2 samples, and detected in at least 5% of the cells of the cluster considered. The package edgeR (version 3.24.3) was used to perform TMM normalization, and to test for differential expression with the Generalized Linear Model (GLM) framework. Genes with a false discovery rate (Benjamini-Hochberg method) lower than 5% were considered differentially expressed. Gene set enrichment analysis was performed with the function camera of the limma package (version 3.44.3) by using the default parameter value of 0.01 for the correlations of genes within gene sets, on gene sets from the Hallmark collection of the Molecular Signature Database (MSigDB, version 6.0). We considered only sets containing more than 5 genes, and gene sets with a false discovery rate (Benjamini-Hochberg method) lower than 20% were considered to be significant. Remaining statistical analysis on the expression dataset analysis and plotting were performed with the R software (version 3.6.0).

### In vitro DEP exposure and Nlrp3 inhibition

Peritoneal macrophages were isolated from wild-type and Nlrp3-/- mice by abdominal lavage. At least 3 to 4 mice were pooled for each experiment. Cells were seeded in a 48-well flat-bottom cell culture plate at a density of 4 × 10^5^/well in RPMI-1640 media. Peritoneal macrophages were primed 6 h with 100ng/mL lipolysaccharide (LPS; Sigma), 30 min prior to exposure to 125 µg/mL DEP or PBS in 250µL/well (31.25 µg/well) overnight (16 h), the cells were pre-incubated with 5µM MCC950 (NLRP3-specific inhibitor; Cat# AG-CR1-3615, Adipogen). At the end of the incubation period, supernatants were collected, and stored at -75^o^C until further use.

### IL-1β antibody treatment

After 4 months of DEP or PBS exposure, mice received 10 mg/kg IL-1β antibody (01BSUR; with the same specificity as canakinumab and as used by other groups [76, 77], Novartis) or isotype control (anti-cyclosporin A, Novartis) i.p. once weekly (MTA Novartis). From week 3 on, mice received once weekly 5 mg/kg.

### Data Availability

scRNA-seq data is available in Gene Expression Omnibus (GEO) under the accession number GSE133406. The data can be accessed using the following link: https://www.ncbi.nlm.nih.gov/geo/query/acc.cgi?acc=GSE133406. Password: ovgjowmsfnevfwr.

### Quantification and statistical analysis

Data are expressed as mean ± SEM. Unpaired Mann-Whitney U test was used for statistical significance (GraphPad Prism, Version 8). p-value < 0.05 was considered statistically significant. GTT data were tested by two-way ANOVA, followed by Sidak’s multiple comparisons test. GTT data show one representative experiment, all other data are pooled.

## Electronic supplementary material

Below is the link to the electronic supplementary material.

Supplementary Material 1

## Data Availability

Data analyzed during the current study are available from the corresponding author upon request. scRNA-seq data is available in Gene Expression Omnibus (GEO) under the accession number GSE133406. The data can be accessed using the following link: https://www.ncbi.nlm.nih.gov/geo/query/acc.cgi?acc=GSE133406 Password: ovgjowmsfnevfwr.

## Abbreviations

ALAT: Alanine transaminase
AP: Alkaline phosphatase
ASAT: Aspartate transaminase
DEP: Diesel exhaust particles
DP: Double positive
EDTA: Ethylenediaminetetraacetic acid
FACS: Fluorescence Activated Cell Sorting
GTT: Glucose tolerance test
HBSS: Hanks’ Balanced Salt solution
IBD: Inflammatory bowel disease
IEL: Intraepithelial lymphocytes
IL-1β: Interleukin-1β
IL-6: Interleukin-6
i.p.: intraperitoneal
NLRP3: NOD-, LRR- and pyrin domain-containing protein 3
PBS: Phosphate-buffered saline
SEM: Standard Error of the Mean
TNF: Tumor necrosis factor
