# Supplementary material for: Diesel Exhaust Particle (DEP)-induced glucose intolerance is driven by an intestinal innate immune response and NLRP3 activation in mice
Source: Part Fibre Toxicol. 2023 Jul 3;20:25. doi: 10.1186/s12989-023-00536-8 (PMC10316612; doi:10.1186/s12989-023-00536-8)
Supplement: Supplementary file 1 — Supplementary Material 1 [file 12989_2023_536_MOESM1_ESM.docx]

**Additional file 1: Figures and Tables**

**Diesel exhaust particle (DEP)-induced glucose intolerance is driven by an**

**intestinal innate immune response and NLRP3 activation in mice**

**Authors:** Angela J. T. Bosch^1^, Theresa V. Rohm^1^, Shefaa AlAsfoor^1^, Andy J. Y. Low^1^, Zora Baumann^1^, Neena Parayil^1^, Faiza Noreen^1,3^, Julien Roux^1,3^, Daniel T. Meier^1^, Claudia Cavelti-Weder^1,4,5*^

**Affiliations:**

^1^Department of Biomedicine, University of Basel, 4031 Basel, Switzerland.

^2^Swiss Institute of Bioinformatics, 4031 Basel, Switzerland.

^3^Clinic of Endocrinology, Diabetes and Metabolism, University Hospital Basel, 4031 Basel, Switzerland.

^4^Department of Endocrinology, Diabetology and Clinical Nutrition, University Hospital Zurich (USZ) and University of Zurich (UZH), Zurich, Switzerland.

**
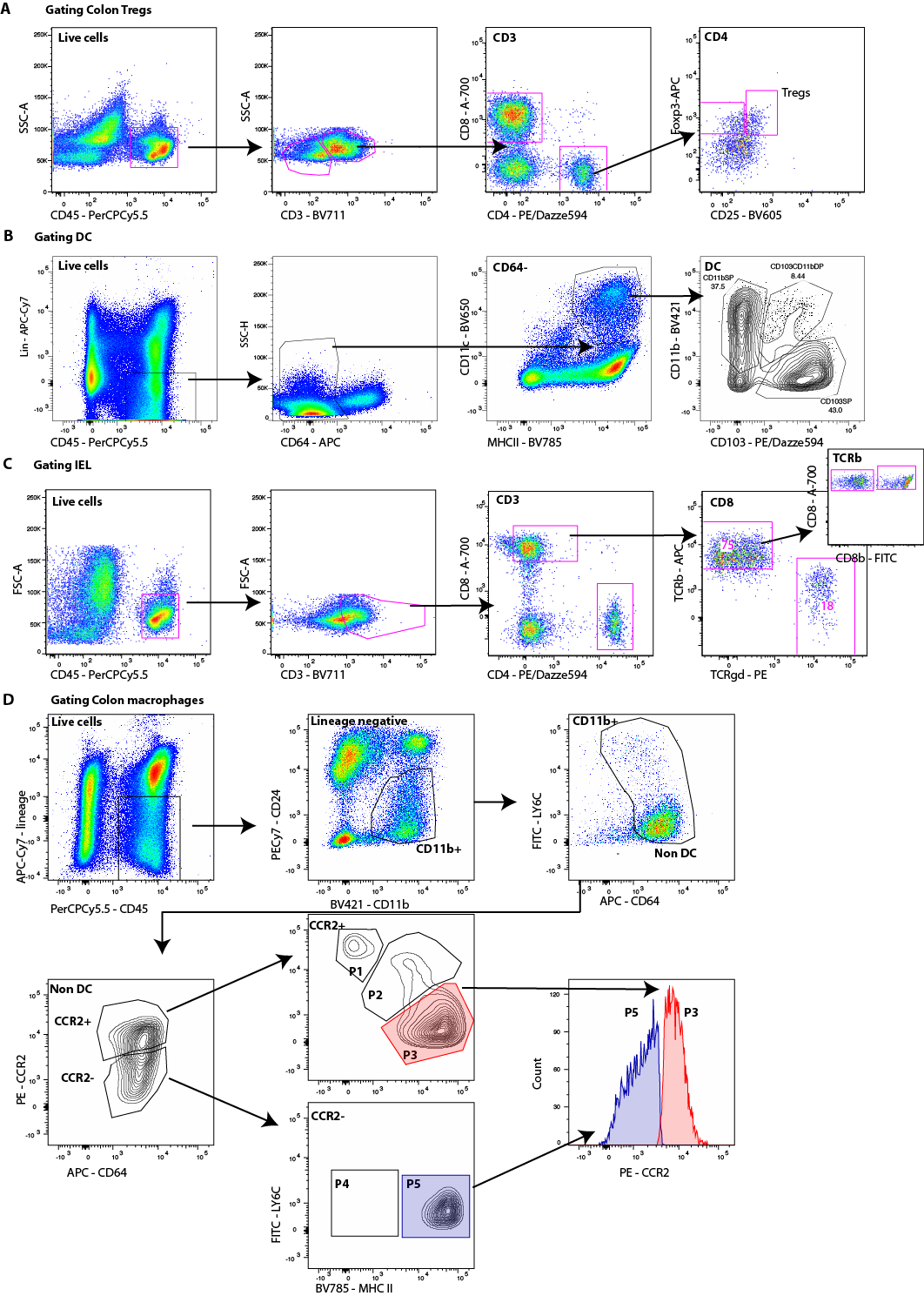
**

**Additional file 1: Figure S1: Gating strategy of colon immune cells.** All gating strategies included a gate for lymphocytes, followed by doublet exclusion and exclusion of dead cells **A** T-cells were defined as CD45^+^/CD3^+^ and either CD8^+^ or CD4^+^, the latter ones were further analyzed for regulatory T cells defined as Foxp3^+^ and CD25^+^. **B** Colon DC were defined as positive for CD45 and negative for lineage markers (lineage cocktail included CD3, CD19, GR1.1 and NK1.1), as well as negative for the expression of CD64. The remaining cells were gated for MHCII and CD11c to identify DC. For determination of subgroups DC were gated for CD103 and CD11b. **C** For intraepithelial lymphocyte (IELs) assessment CD45^+^ cells were gated for CD3^+^ cells and further divided into CD4^+^ and CD8^+^ T-cells, CD8^+^ T-cells were further gated for expression of TCRαβ, TCRγδ, CD8αα, and CD8αβ. **D** To identify colon macrophages, cells isolated from the lamina propria were first gated for the expression of CD45 and the absence of lineage markers (lineage cocktail included CD3, CD19, GR1.1 and NK1.1). The cells were further gated for CD11b and absence of CD24 expression. In a subsequent gate, cells that were double negative for Ly6C and CD64 were excluded, and the remaining cells were further gated for the expression of CCR2. CCR2+ colon macrophages were defined as inflammatory macrophages, this subset could be further divided into 3 different subpopulations P1-P3, where the expression of Ly6C is lost and MHCII expression acquired. The CCR2- population was defined as anti-inflammatory colon macrophages with two subpopulations P4 and P5. In respect to the expression of Ly6C and MHCII P3 and P5 could not be separated, however the two populations clearly differ in their CCR2 expression.

**Additional file 1: Figure S2: Ahrr methylation and gene expression are unchanged upon diesel exposure.** **A** The UCSC browser view showing Ahrr. In green is CpG island track. Primers were designed to detect methylation levels at both CpG islands in Ahrr gene. Schematic shows two sequencing regions, identifying methylation status of 26CpGs (Region 1: 10CpGs; Region 2: 16CpGs) with Ahrr sequencing primer positions (arrows), CpGs analyzed (black vertical lines), CpGs not analyzed (grey vertical lines). **B** Left: scatter plot showing mean methylation levels for each CpG in PBS (controls, n=6) and DEP exposed mice (n=6). Each circle represents one CpG. Right: boxplots of the resultant methylation levels on all 26CpGs per mouse. Shown are median (line) and mean (black circle). **C** Ahrr gene expression in colon of mice exposed for 9 months with PBS or DEP. A,B represent one experiment, C pooled data from two independent experiments. Data are presented as mean±SEM and compared by a two-tailed, unpaired with Mann-Whitney U test.


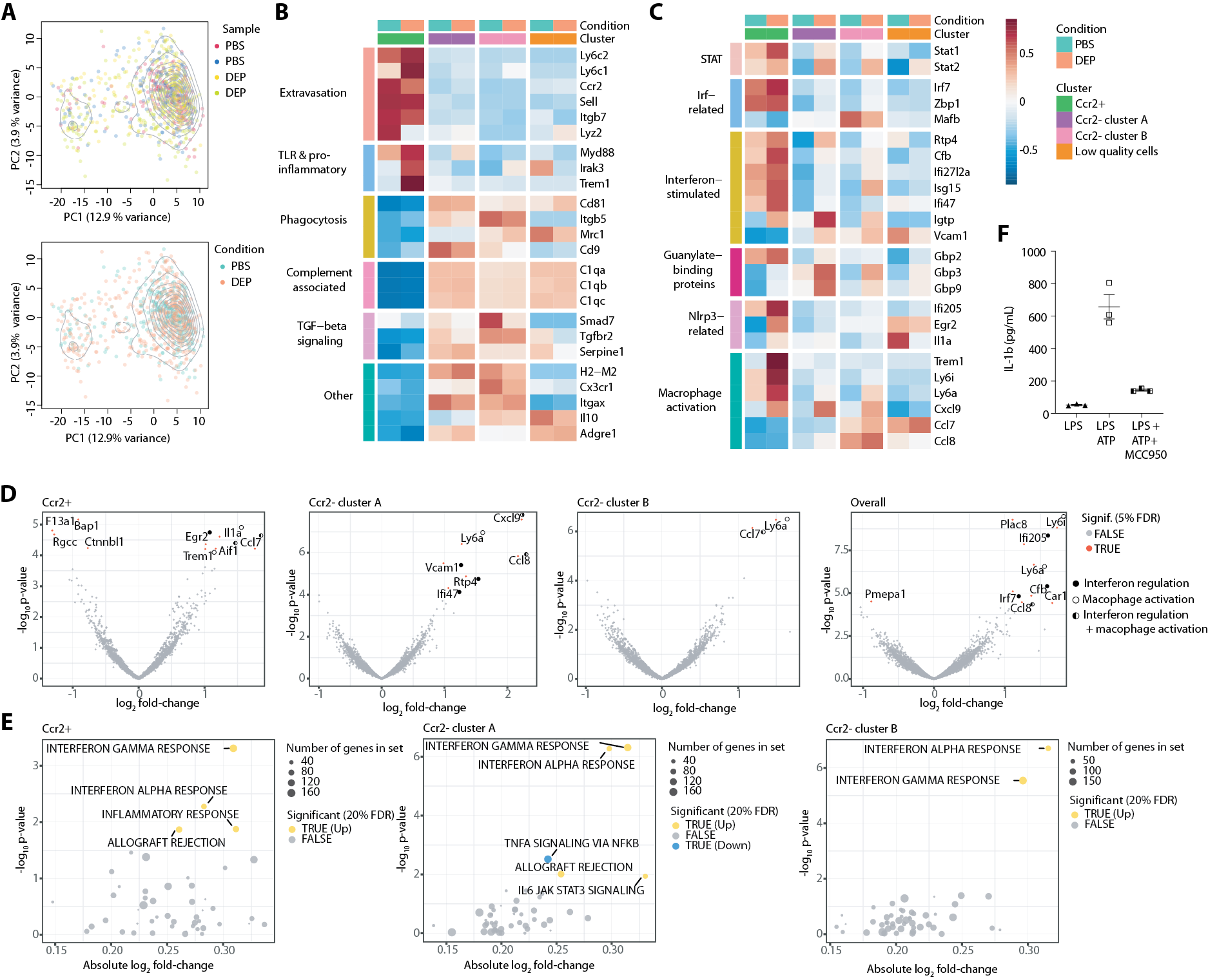


**Additional file 1: Figure S3: Differences in genes expression in mice exposed to diesel. A** Principal component analysis of colon macrophages showing *Ccr2*^+^ and *Ccr2*^-^ subpopulations (colors represent different clusters; contour lines indicate cell density) for each sample and per condition (diesel exhaust particles (DEP), phosphate-buffered saline (PBS)). **B** Functionally defined subsets of selected marker genes up- or downregulated during differentiation from *Ccr2*^+^ to *Ccr2*^-^ colon macrophages of mice exposed to DEP or PBS. **C** Heatmap of averaged expression of selected genes from interferon regulation, inflammasome regulation, and macrophage activation in mice exposed to DEP or PBS across clusters and conditions. **D** Volcano plots for the differential expression analysis of FACS sorted colon macrophages from mice exposed to DEP or PBS, from an analysis stratified into the different clusters. Significant genes at an FDR of 5% are shown in red and labelled; non-significant genes are shown in grey. Closed circles indicate genes related to interferon regulation, open circles genes related to macrophage activation, half-open circles to both. **E** Effects of Diesel on MSigDB Hallmark Pathways in *Ccr2*^+^ and *Ccr2*^-^ Colon Macrophages. MSigDB Hallmark pathways that were up- (yellow) or down-regulated (blue) in colon macrophages upon exposure to diesel, from an analysis stratified into *Ccr2*^+^ and *Ccr2*^-^ clusters. Data were obtained from one experiment with a group size of 2 animals per group. **F** Protein expression of IL-1β in the supernatant of peritoneal macrophages isolated from wild-type mice *in vitro* stimulated with LPS±ATP±the NLRP3-inhibitor MCC950. DEP: diesel exhaust particles, PBS: phosphate-buffered saline (control).

**Additional file 1: Tables**

| REAGENT or RESOURCE | SOURCE | IDENTIFIER |
| --- | --- | --- |
| Antibodies | | |
| Anti-mouse CD16/32 (93) | Biolegend | Cat#101321; RRID: [AB_2103871](http://antibodyregistry.org/AB_2103871) |
| Anti-mouse CD11c (N418) BV650 | Biolegend | Cat#117339; RRID: [AB_2562414](http://antibodyregistry.org/AB_2562414) |
| Anti-mouse CD11c (N418) PE-Cy7 | Biolegend | Cat#117318; RRID: [AB_493568](http://antibodyregistry.org/AB_493568) |
| Anti-mouse CD11b (M1/70) BV421 | Biolegend | Cat#101236; RRID: [AB_11203704](http://antibodyregistry.org/AB_11203704) |
| Anti-mouse CD45 (30-F11) PerCP-Cy5.5 | Biolegend | Cat#103131; RRID: [AB_893344](http://antibodyregistry.org/AB_893344) |
| Anti-mouse I-A/I-E (M5/114.15.2) BV785 | Biolegend | Cat#107645; RRID: [AB_2565977](http://antibodyregistry.org/AB_2565977) |
| Anti-mouse Ly6C (HK1.4) FITC | Biolegend | Cat#128005; RRID: [AB_1186134](http://antibodyregistry.org/AB_1186134) |
| Anti-mouse CCR2 (475301) PE | R&D Systems | Cat#FAB5538P; RRID: [AB_10718414](http://antibodyregistry.org/AB_10718414) |
| Anti-mouse CD103 (2E7) PE-Dazzle594 | Biolegend | Cat#121430; RRID: [AB_2566493](http://antibodyregistry.org/AB_2566493) |
| Anti-mouse CD24 (M1/69) PE-Cy7 | Biolegend | Cat#101821; RRID: [AB_756047](http://antibodyregistry.org/AB_756047) |
| Anti-mouse CD64 (X54-5/7.1) APC | Biolegend | Cat#139306; RRID: [AB_11219391](http://antibodyregistry.org/AB_11219391) |
| Anti-mouse CD3 (145-2C11) APC-Cy7 | Biolegend | Cat#100330; RRID: [AB_1877170](http://antibodyregistry.org/AB_1877170); |
| Anti-mouse Nk1.1 (PK136) APC-Cy7 | Biolegend | Cat#108723; RRID: [AB_830870](http://antibodyregistry.org/AB_830870) |
| Anti-mouse CD19 (6D5) APC-Cy7 | Biolegend | Cat#115530; RRID: [AB_830707](http://antibodyregistry.org/AB_830707) |
| Anti-mouse Siglec F (E50-2440) BV510 | BD Biosciences | Cat#740158; RRID: [AB_2739911](http://antibodyregistry.org/AB_2739911) |
| Anti-mouse F4/80 (BM8) PE | Biolegend | Cat#123110; RRID: [AB_893486](http://antibodyregistry.org/AB_893486) |
| Anti-mouse CD206 (C068C2) A647 | Biolegend | Cat#141712; RRID: [AB_10900420](http://antibodyregistry.org/AB_10900420) |
| Anti-mouse CD3 (17A2) BV711 | Biolegend | Cat#100241; [RRID:AB_2563945](https://antibodyregistry.org/search.php?q=AB_2563945) |
| Anti-mouse TCRβ (H57-597) APC | Biolegend | Cat#109211;[RRID:AB_313434](https://antibodyregistry.org/search.php?q=AB_313434) |
| Anti-mouse TCRγδ (GL3) PE | Biolegend | Cat#118107; [RRID:AB_313831](https://antibodyregistry.org/search.php?q=AB_313831) |
| Anti-mouse CD8a (53-6.7) A700 | Biolegend | Cat#100729;[RRID:AB_493702](https://antibodyregistry.org/search.php?q=AB_493702) |
| Anti-mouse CD8b (YTS156.7.7) FITC | Biolegend | Cat#126605; [RRID:AB_961293](https://antibodyregistry.org/search.php?q=AB_961293) |
| Anti-mouse CD4 (GK1.5) PE-Dazzle 594 | Biolegend | Cat#100456; [RRID:AB_2565845](https://antibodyregistry.org/search.php?q=AB_2565845) |
| Anti-mouse CD25 (PC61) BV605 | Biolegend | Cat#102035; [RRID:AB_11126977](https://antibodyregistry.org/search.php?q=AB_11126977) |
| Anti-mouse Foxp3 (FJK-16s) APC | Thermo Fisher Scientific | Cat#17-5773-82; [RRID:AB_469457](https://antibodyregistry.org/search?q=17-5773-82) |
| Anti-mouse Rorγ (Q31-378) BV786 | BD Biosciences | Cat#564723; RRID:AB_273891 |
| anti-GATA3 (TWAJ) A488 | Thermo Fisher Scientific | Cat#52-9966-43; [RRID:AB_2574493](file:///Users/angela/Library/Containers/com.microsoft.Word/Data/Desktop/STM_2020_08_24/Nature_metabolism/AB_2574493) |
| **Chemicals, Peptides, and Recombinant Proteins** | | |
| Diesel exhaust particles (DEP) | National Institute of Standards and Technologies/Sigma | Cat# NIST1650B |
| Collagenase IV | Worthington | Cat# LS004189 |
| Collagenase VIII | Sigma-Aldrich | Cat# C2139 |
| DNase 1 | Roche | Cat# 11284932001 |
| Percoll | GE Healthcare | Cat# GE17-0891-01 |
| PLX5622 (CFS1R inhibitor) | Plexxikon | MTA |
| anti-IL-1β (01BSUR) | Novartis | MTA |
| **Critical Commercial Assays** | | |
| Mouse/rat insulin kit | MesoScale Diagnostics | Cat#K152BZC |
| V-Plex custom mouse cytokine proinflammatory panel 1 mouse TNF-α, mouse IL-6 and mouse IL-1β | MesoScale Diagnostics | Cat#K15048 |
| Foxp3 staining Kit | Thermo Fisher Scientific | Cat# 00-5523-00 |
| NucleoSpin RNA kit | Macherey Nagel | Cat# 740955 |
| RNeasy Plus Universal Mini kit | Qiagen | Cat# 73404 |
| GoScript^TM^ | Promega | Cat# A5003 |
| GoTaq qPCR Master Mix | Promega | Cat# A4472919 |
| **Deposited Data** | | |
| scRNA-seq Data | This paper | GSE133406 |
| **Experimental Models: Organisms/Strains** | | |
| Mouse, C57BL/6NCrl | Charles River laboratories | RRID:IMSR_CRL:027 |
| Mouse, B6.129S4-Ccr2^tm1Ifc^/J | The Jackson laboratory | RRID:IMSR_JAX:004999 |
| Mouse: C57BL/6J | University of Basel | RRID:IMSR_JAX:000664 |
| Mouse, B6.129S6-Rag2^tm1Fwa^ | Taconic biosciences | RRID:IMSR_TAC:RAGN12 |
| Mouse, B6-Nlrp3<tm1Tsc>/N | Prof M. Donath, University of Basel | N/A |
| **Oligonucleotides** | | |
| Primers for qPCR -> Suppl. Table 2 | Microsynth | N/A |
| **Software and Algorithms** | | |
| Flow jo (version 9.9 or higher) | Becton Dickinson & Company (BD) | https://flowjo.com |
| BD FACS Diva (version 8.0.1) | Becton Dickinson & Company (BD) | <https://www.bdbiosciences.com/en-us/instruments/research-instruments/research-software/flow-cytometry-acquisition/facsdiva-software> |
| Prism 8 | GraphPad Software, LLc. | https://www.graphpad.com |
| R version 3.6 | The R Foundation | <https://www.r-project.org> |

Additional file 1: Table S1. Reagent and resource table.

| **Gene** | **Forward Primer** | **Reverse Primer** |
| --- | --- | --- |
| **Housekeeping genes** | | |
| ***B2m*** | 5′ TTCTGGTGCTTGTCTCACTGA | 5′ CAGTATGTTCGGCTTCCCATTC |
| ***Ppia*** | 5′ GAGCTGTTTGCAGACAAAGTTC | 5′ CCCTGGCACATGAATCCTGG |
| **Inflammation markers** | | |
| ***Tnf*** | 5′ ACTGAACTTCGGGGTGATCG | 5′ TGAGGGTCTGGGCCATAGAA |
| ***Il6*** | 5′ GGATACCACTCCCAACAGACCT | 5′ GCCATTGCACAACTCTTTTCTC |
| ***Il1b*** | 5′ GCAACTGTTCCTGAACTCAACT | 5′ ATCTTTTGGGGTCCGTCAACT |
| ***Cxcl1 (KC)*** | 5′ CTGGGATTCACCTCAAGAACATC | 5′ CAGGGTCAAGGCAAGCCTC |
| ***Il10*** | 5′ AGGCGCTGTCATCGATTTCTC | 5′ GCCTTGTAGACACCTTGGTCTT |
| ***Il18*** | 5′TCTTGCGTCAACTTCAAGGA | 5′GTGAAGTCGGCCAAAGTTGT |
| ***Il22*** | 5′TTG AGG TGT CCA ACT TCC AGC A | 5′AGC CGG ACG TCT GTG TTG TTA |
| ***Tgfb1*** | 5′CTCTCCACCTGCAAGACCAT | 5′CGAGCCTTAGTTTGGACAGG |
| ***Tgfb2*** | 5′GAAATACGCCCAAGATCGAA | 5′TGTCACCGTGATTTTCGTGT |
| ***Ifng*** | 5′GTCTCTTCTTGGATATCTGGAGGAACT | 5′GTAGTAATCAGGTGTGATTCAATGACGC |
| ***Il17*** | 5′ATC AGG ACG CGC AAA CAT GA | 5′TTG GAC ACG CTG AGC TTT GA |
| **Immune cells** | | |
| ***Cd68*** | 5′ GCAGCACAGTGGACATTCAT | 5′ AGAGAAACATGGCCC GAAGT |
| ***Adgre1 (Emr1)*** | 5′ GCC CAG GAGTGGAATGTCAA | 5′ CAGACACTCATCAACATCTGCG |
| ***Ly6c1*** | 5’GCA GTG CTA CGA GTG CTA TGG | 5’ACT GAC GGG TCT TTA GTT TCC TT |
| **Beta-cell identity** | | |
| ***Pdx1*** | 5′CCC CAG TTT ACA AGC TCG CT | 5′CTC GGT TCC ATT CGG GAA AGG |
| ***Foxo1*** | 5′GTA CGC CGA CCT CAT CAC CA | 5′TGC TGT CGC CCT TAT CCT TG |
| ***Ins2*** | 5′CCC TGC TGG CCC TGC TCT T | 5′AGG TCT GAA GGT CAC CTG CT |

**Additional file 1: Table S2. Primers sequences used for quantitative real time-PCR.**

| **Gene** | **Primer** | **Sequence (5’-3’)** |
| --- | --- | --- |
| ***Ahrr*** | Forward1 | TGGGATTTGGTTTTAGATTAGAGAAG |
|  | Reverse1 | GCCCCCGCCCGAACACCCTATCCAAACACTCAAAC |
|  | Forward-nested1 | GTTTTAGATTAGAGAAGGAAAGTTTTAT |
|  | Sequencing-S1 | GAAGGAAAGTTTTATTTTAGT |
|  |  |  |
|  | Forward2 | AGTTTAAGTTGAGTTTTTGGGAT |
|  | Reverse2 | GCCCCCGCCCGAATCTAAACCCCTAACCAATTTACC |
|  | Sequencing-S2 | GTTGAGTTTTTGGGATTT |
|  |  |  |
| Universal primer |  | Biotin-GCCCCCGCCCG |

Additional file 1: Table S3. Pyrosequencing primer sequences. Sequence underlined represents the 11-base tag.
